# Study on Preparation and Antibacterial Property of DOMA-SBMA Copolymer Coatings on Stainless Steel Surfaces

**DOI:** 10.3390/ma19020242

**Published:** 2026-01-07

**Authors:** Fei Wan, Linlin Zhang, Chao Feng, Wenwen Yan, Andreas Hermann Gerdes, Ruixuan Tong, Zhengyang Zhou

**Affiliations:** Materials Science and Engineering, Civil Engineering, Qingdao University of Technology, Qingdao 266520, China; wanfei@qut.edu.cn (F.W.); 17863953125@163.com (L.Z.); yanwenwen_2009@163.com (W.Y.); andreas.gerdes@kit.edu (A.H.G.); a19862223407@163.com (R.T.); 17860966066@163.com (Z.Z.)

**Keywords:** zwitterionic polymers, surface modification, antibacterial coating, polydopamine

## Abstract

A combination of surface wettability and antibacterial performance is highly imperative for construction of antibacterial coatings. In this study, motivated by the antibacterial properties of zwitterionic polymer, mussel-inspired adhesion, and the “grafting to”, a novel DOMA-SBMA copolymer with adhesion and wettability is developed for constructing a bacteriostatic surface. Specifically, the antibacterial coating is prepared via free radical polymerization and grafting to methods on the surface of stainless steel, and characterized by SCA, FTIR, XPS, SEM, and AFM to confirm the modification process. Antibacterial activity evaluation using *Staphylococcus aureus* (*S. aureus*) and *Escherichia coli* (*E. coli*) shows that the coating presents satisfactory antibacterial performance. The results showed that DOMA-SBMA coating is enough for antibacterial application, with high antibacterial efficiency against *E. coli* (92.2%) and *S. aureus* (95.0%). In summary, the bioinspired coating developed here may improve the stability of zwitterionic coatings and provides a simple preparation strategy for constructing antibacterial coatings.

## 1. Introduction

Bacteria, while being minute organisms, present a significant threat to human health and economic stability. More than 4000 fouling species have been found in the maritime environment [1]. This encompasses microorganisms such as bacteria, charophyte spores, and diatoms, in addition to larger species including mussels, bryozoans, barnacles, and algae. When affixed to artificial structures like vessels and oil platforms, these fouling organisms have considerable adverse impacts on marine operations. The effects include obstructed drainage pipes, diminished ship velocity, expedited hull deterioration, heightened fuel consumption, and higher greenhouse gas emissions. Annually, significant resources are allocated for the cleaning of ship exteriors and the upkeep of marine infrastructures. Nosocomial infections pose a prevalent health hazard, exhibiting fatality rates above 15% in developing countries [2]. The prevention of detrimental bacterial proliferation and buildup depends on the application of antimicrobial coatings that impart antibacterial capabilities to material surfaces.

Antimicrobial coatings are primarily classified into three groups according to their modes of action: release-based antimicrobial coatings [3,4,5,6], contact-active coatings [7,8,9,10], and anti-adhesive coatings [11,12,13,14,15]. Release-based coatings function by continually leaching active biocidal chemicals into the environment. These coatings are effective in the short term. Nonetheless, their utilization raises particular environmental safety issues. Heavy metal compounds, including copper(I) oxide, tributyltin, and mercury oxide, can bioaccumulate in aquatic creatures such as fish and shellfish. This accumulation can result in biological anomalies, population loss, and seawater contamination, hence posing long-term threats to marine ecosystems [16]. Conversely, contact-active coatings immobilize bacteria upon contact through surface-bound physicochemical mechanisms, such as cationic charge disruption or photocatalytic oxidation. Furthermore, anti-adhesive antimicrobial coatings impede early bacterial adhesion by establishing a physical or energetic barrier, exemplified by low-surface-energy or zwitterionic materials. Although release-type antimicrobial coatings are effective, their environmental risks highlight the necessity for the development of sustainable, eco-friendly alternatives. Amphoteric materials are a category of biocompatible chemicals that possess both positively and negatively charged functional groups. They exhibit superior lubricity, antifouling characteristics, and exceptional biocompatibility, positioning them as a central focus in contemporary research on environmentally sustainable marine antifouling coatings [17,18,19]. Zwitterionic structures that have been extensively researched include phosphatidylcholine, carboxybetaine, and sulfobetaine [20,21,22,23]. Zhang et al. [24] utilized a one-pot procedure to graft zwitterionic esters and capsaicin polymers onto a polydimethylsiloxane (PDMS) network, resulting in the development of multifunctional marine antifouling coatings with exceptional anti-adhesive characteristics. Xu et al. [25] synthesized a zwitterionically functionalized polylaccinol antifouling coating by amalgamating the zwitterionic chemicals laccinol and sulfonated betaine. This coating exhibited substantial inhibitory effects against both bacteria and microalgae while demonstrating exceptional environmental sustainability.

The sulfonic acid and quaternary ammonium groups in the molecular structure of sulfobetaine methacrylate (SBMA) create a highly efficient hydration layer, making it a widely utilized zwitterionic molecule. This hydration layer significantly inhibits the adhesion of marine fouling organisms. Moreover, sulfonated betaine compounds are biodegradable, providing superior biocompatibility and environmental safety [26,27,28]. Nevertheless, in real applications, the coating may delaminate due to friction or hydrolysis, leading to reduced antibacterial effectiveness. Researchers have extensively explored various synthetic methods for preparing antimicrobial coatings, including physical mixing (simple blending of active agents with coating materials before application), physical coating (direct application of antimicrobial substances onto surfaces), “grafting from” and “grafting to” [29,30,31]. However, coatings prepared via physical mixing and physical coating exhibit poor surface/interface stability during use, often leading to peeling. In contrast, “grafting from” is a technique in which chemical groups called radicals are generated directly on the surface; these radicals act as initiation points for polymerization after an initiator is chemically attached to the surface. Monomers—small, reactive molecules—then link together, growing polymer chains directly from the surface to form hydrophilic (water-attracting) polymer macromolecules. This method increases the amount of modifier on the surface, enabling the formation of uniform, stable polymer coatings on solids. Nevertheless, its process is relatively complex, with reaction conditions that are challenging to control and often require an oxygen-free environment. Additionally, the resulting modified surfaces are difficult to analyze. Alternatively, “grafting to,” the most common coating preparation method, involves attaching preformed polymer chains (long molecular chains with chemically reactive ends) to substrate surfaces that have compatible reactive groups, allowing the polymers to bind to the surface.

Strong adherence and exceptional antibacterial characteristics are essential conditions for the efficacy of antimicrobial coatings. To improve polymer stability on material surfaces, zwitterionic polymers must contain robust anchoring groups. Motivated by the adhesion mechanism of mussel byssus threads, researchers have created a range of catechol-infused polymers that can securely bond to diverse substrate surfaces [32,33,34,35]. Research demonstrates that their remarkable adhesive qualities arise from the presence of 3,4-dihydroxyphenylalanine (DOPA) [36,37]. Catechols, via catechol chemistry, function as pivotal groups on various substrates, facilitating mechanisms including metal coordination, hydrogen bonding, π-π interactions, cation-π interactions, borate-catechol complexation, hydrophobic interactions, Schiff base reactions, Michael addition reactions, and covalent bonding [38]. Catechol, a prevalent surface anchoring group, can be covalently linked to zwitterionic polymer chains through controlled radical polymerization, facilitating stable polymer adhesion to surfaces [39,40].

This study reports the synthesis of methacryloyldopamine (DOMA) via the acylation of dopamine hydrochloride with methacryloyl chloride. A copolymer was produced using radical polymerization of DOMA and SBMA, and a distinctive DOMA-SBMA antibacterial coating was effectively applied to stainless steel surfaces utilizing a “grafting-to” technique. The new ampholytic polymer was analyzed using static contact angle (SCA), Fourier transform infrared spectroscopy (FTIR), X-ray photoelectron spectroscopy (XPS), scanning electron microscopy (SEM), atomic force microscopy (AFM), and antibacterial efficacy. The preparation procedure for this coating is uncomplicated and can improve the stability of zwitterionic coatings, offering a direct strategy for developing antibacterial coatings.

## 2. Materials and Methods

### 2.1. Materials

2,2′-Azobisisobutyronitrile (AIBN, 98%), and dopamine hydrochloride (DA, 98.5%) were purchased from Shanghai Macklin Biochemical Technology Co., Ltd. (Shanghai, China). N,N-Dimethylformamide (DMF, 99.9%), methanol-d_4_ (MeOD-d_4_) and lithium bromide (99.9%) were obtained from Sigma-Aldrich (St. Louis, MO, USA). Methacryloyl chloride (90%), ethyl acetate (99.5%), hydrochloric acid (AR, 36.0−38.0%), anhydrous sodium sulfate (AR, 99.0%), and sulfobetaine methacrylate (SBMA, 98%) were purchased from Aladdin Reagent Co., Ltd. (Shanghai, China). Methanol (99.5%), Ethanol absolute (99.5%) and sodium borate (AR, 99.5%) were purchased from Sinopharm Chemical Reagent Co., Ltd. (Shanghai, China). Sodium carbonate (AR, 99.8%) was purchased from Tianjin Chemical Reagent Factory (Tianjin, China). Nitrogen gas (N_2_) was purchased from Qingdao Ludong Gas Co., Ltd. (Qingdao, China). Stainless steel sheets (316L) were acquired from Jinchen Stainless Steel Products Co., Ltd. (Dongguan, China), and 400 grit sandpaper was obtained from Huayan Abrasives Co., Ltd. (Shanghai, China). The bacterial strains used in this study were *S. aureus* ATCC25923, *E. coli* ATCC25922, obtained from the Institute of Marine Energy, Chinese Academy of Sciences (Qingdao, China).

### 2.2. Characterization

Fourier transform IR spectroscopy (FTIR, Nicolet 6700, Thermo Fisher Scientific, Waltham, MA, USA) analysis was carried out in the region of 4000–400 cm^−1^ for 32 scans. ^1^H nuclear magnetic resonance (^1^H NMR, DRX-600, Bruker BioSpin GmbH, Rheinstetten, Germany) spectroscopy was carried out with all measurements performed at room temperature using MeOD-d_4_ as the solvent.

The number-average molecular weight (M_n_) and polydispersity index (PDI, M_w_/M_n_) of the samples were determined by gel permeation chromatography (GPC). All GPC measurements were performed on a Waters 1525 system equipped with a Waters 2414 detector (Waters Corporation, Milford, MA, USA) and an Agilent PLgel 5 μm MIXED-C column (Agilent Technologies, Santa Clara, CA, USA). GPC grade DMF stabilized with 0.1 M lithium bromide was used as the eluent at a flow rate of 1 mL/min, and the analysis was conducted at 50 °C. Calibration was carried out using poly(methyl methacrylate) standards.

Coating thickness was determined via spectroscopic ellipsometry (SE, UVISEL, HORIBA Jobin Yvon, Longjumeau, France) with measurements performed at a fixed incident angle of 70°, and the results were expressed as mean ± standard deviation (SD, n = 3) of three parallel samples.

The surface wettability of the composite coating was studied using an optical contact angle measuring instrument (OCA, SDC-200, Shengding Precision Instruments Co., Ltd., Dongguan, China) on unmodified 316L stainless steel surfaces. Under a controlled temperature condition of 21 °C, adding a 2 μL drop of deionized water on 5 different surface areas. Three measurements were performed for each sample, and the average value was calculated.

The elemental composition and chemical states were analyzed by X-ray photoelectron spectroscopy (XPS, Escalab 250Xi, Thermo Fisher Scientific, Waltham, MA, USA), using Al Kα as the excitation source. XPS data were analyzed using Thermo Avantage software (Version 6.9, Thermo Fisher Scientific, Waltham, MA, USA), and thse high-resolution spectra of each element were fitted.

The micromorphology of the composite coating was studied using scanning electron microscope (SEM, Sigma300, DEU, Carl Zeiss AG, Oberkochen, Germany) and atomic force microscope (AFM, Dimension Icon, Bruker Corporation, Santa Barbara, CA, USA). The scanning frequency was set to 1 Hz and the sampling resolution to 256. A 5 μm × 5 μm area was selected for scanning on each sample surface. The obtained data were analyzed using Gwyddion software (Version 2.63, Czech Metrology Institute, Brno, Czech Republic) to determine the average value of surface roughness (Rq).

The antibacterial efficacy of the DOMA-SBMA coating was evaluated by using *E. coli* and *S. aureus*. The optical micrograph of the DOMA-SBMA composite coating was observed through a fluorescence microscope (FL, DMI8, DEU). Briefly, a 20 μL aliquot of SYTO-9 nucleic acid stain (2 μL/mL) was applied to cover the central area of each sample. For each sample, at least three random fields of view were captured and the adhering bacteria on the surface of the sample were statistically analyzed using the threshold analysis method in ImageJ (Version 1.50b, National Institutes of Health, Bethesda, MD, USA) [41]. The obtained experimental data were subjected to one-way analysis of variance (ANOVA) with the IBM SPSS Statistics software (Version 26.0, IBM Corporation, Armonk, NY, USA). Statistical significance was determined at the level of *p* < 0.05.

### 2.3. Preparation of DOMA-SBMA Coating

DOMA was prepared according to Reference [42], and the detailed preparation procedure is outlined below: A sodium borate solution (10 mmol, 3.83 g) prepared by dissolving the solid in 100 mL ultrapure water was transferred into a 250 mL round-bottomed flask, and N_2_ was continuously purged through the solution for 10 min. Then, DA (1.9 g, 10 mmol) was added to the flask, and the pH was adjusted to 9–10 with sodium carbonate (3.99 g, 32 mmol). The resulting solution was then cooled in an ice-water bath. After that, methacryloyl chloride (1.05 g, 10 mmol) was added, and the mixture was further stirred at 25 °C for 24 h. Finally, the product was obtained by adjusting the pH of the mixture to 2 with 6 M hydrochloric acid, after which the product was extracted with ethyl acetate, dried over anhydrous sodium sulfate, and concentrated via rotary evaporation. The final product was a brown solid DOMA (0.25 g, yield 11.24%).

A series of zwitterionic polymers were synthesized by free radical polymerization with different monomer molar ratios of DOMA:SBMA = 4:6, 2:8, and 1:9. For a typical synthesis procedure, taking the DOMA-SBMA_2/8_ sample as an example, DOMA (0.2658 g, 1.2 mmol), SBMA (1.341 g, 4.8 mmol), and a predetermined amount of AIBN (0.06 mmol) were dissolved in 10 mL of a methanol/water mixed solvent (*v*/*v* = 1:1). The polymer solution was then transferred to a dry Schlenk tube. The reaction system was deoxygenated by bubbling N_2_ gas for 30 min, followed by stirring at 45 °C for 24 h. After cooling to room temperature, the crude product was obtained by pouring the reaction mixture into excess methanol. To remove unreacted monomers and residual initiator, the crude product was further purified via three rounds of reprecipitation from methanol. The synthesized zwitterionic polymers were designated as DOMA-SBMA_4/6_, DOMA-SBMA_2/8_, and DOMA-SBMA_1/9_.

The 316L stainless steel was cut into square pieces (10 mm × 10 mm × 3 mm), repeatedly polished with 400-grit sandpaper, rinsed with deionized water and ethanol, and then dried in air for later use. The surface of 316L stainless steel was pretreated with oxygen plasma using a quartz plasma cleaner (PT-2SM, Shenzhen Sanhe Boda Electromechanical Technology Co., Ltd., Shenzhen, China) for 20 min to activate it. The 316L stainless steel samples were immersed in aqueous solutions of zwitterionic copolymer at concentrations of 5 mg/mL, 10 mg/mL, 15 mg/mL, 20 mg/mL, and 25 mg/mL, respectively, and reacted for 12 h to bond the copolymer onto the stainless steel surface. Then the samples were taken out, thoroughly rinsed with deionized water and ethanol, and dried under nitrogen atmosphere.

### 2.4. Cell Adhesion

Under sterile conditions, *S. aureus* and *E. coli* were inoculated into 20 mL LB liquid medium using a sterile inoculating loop (14-959-101, Thermo Fisher Scientific, Waltham, MA, USA). The bacterial culture was incubated in a 37 °C constant-temperature incubator for 24 h. Following incubation, the culture was subjected to centrifugation at 5000 rpm and 4 °C for 8 min; the supernatant was discarded, and the bacterial pellet was resuspended in sterile PBS. This procedure was repeated three times to eliminate residual LB medium. Finally, the bacterial pellet was resuspended again and diluted with sterile PBS to a final concentration of approximately 1 × 10^6^ CFU/mL. 316L stainless steel and modified samples were placed in 24-well plates, UV-sterilized for 30 min in a sterile biosafety cabinet, and rinsed thoroughly with PBS to remove suspended cells. The PBS was then replaced with freshly prepared bacterial suspension, and the samples were incubated at 37 °C under saturated humidity with incubation time points set at 1, 3, and 7 days. The bacterial suspension was refreshed daily to maintain system stability. After incubation, the samples were gently rinsed with PBS to remove loosely adherent bacteria and transferred to new 24-well plates for subsequent experiments.

## 3. Results and Discussion

### 3.1. Synthesis and Characterization of DOMA-SBMA Copolymer

**Figure 1:**
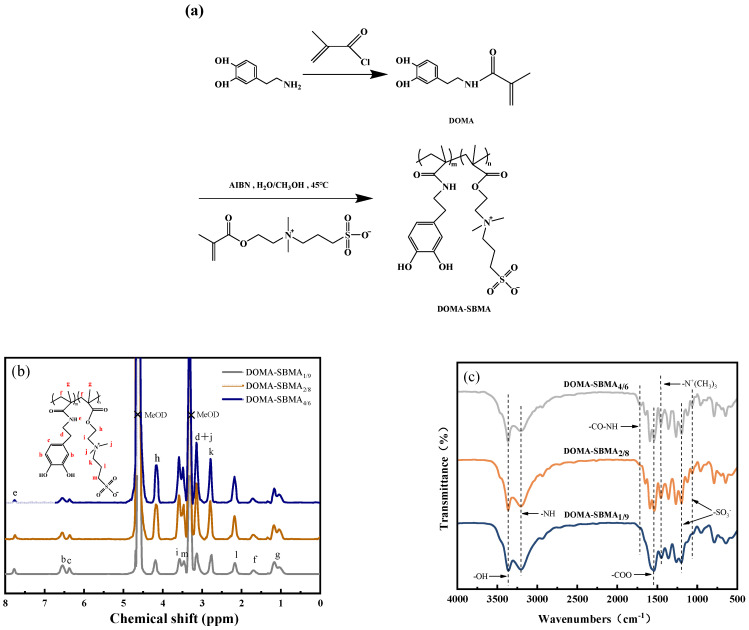
(**a**) Scheme of the preparation of DOMA-SBMA copolymer; (**b**) ^1^H NMR spectra of the DOMA-SBMA copolymer; (**c**) FTIR spectra of DOMA-SBMA copolymer.

FTIR spectra of DOMA-SBMA_4/6_, DOMA-SBMA_2/8_, and DOMA-SBMA_1/9_ are presented in Figure 1c. The broad band at 3350 cm^−1^ is the characteristic peak of –OH. The stretching vibrations of –CO–NH and –NH are observed at 1700 cm^−1^ and 3200 cm^−1^. The stretching vibrations of –COO and –N^+^(CH_3_)_3_ are located at 1610 cm^−1^ and 1460 cm^−1^ in the samples. The symmetrical and asymmetrical stretching vibrations of –SO^3−^ are observed at 1055 cm^−1^ and 1190 cm^−1^.

These results indicate that DOMA and SBMA were copolymerized successfully. As shown in Table 1, with the decrease in DOMA content in the feed formulation, both the PDI and M_n_ of the DOMA-SBMA copolymers exhibited an increasing trend. All copolymers displayed a PDI ranging from 1.60 to 1.66, which is typical for free radical polymerization systems. The M_n_ of all copolymers exceeded 22,000 g/mol, with the Mn ranging from 22,300 to 26,900 g/mol. Notably, the copolymer molecular weight decreased with the reduction in DOMA content, which can be attributed to the role of catechol groups as free radical scavengers during free radical polymerization. Specifically, these groups terminate the growth of polymer chains, thereby reducing the final molecular weight.

Figure 1b presents the ^1^H NMR spectrum of the DOMA-SBMA copolymer with corresponding peak assignments. The peaks at δ = 4.70 and 3.30 ppm are solvent peaks used for MeOD-d_4_. The molar ratio of DOMA to SBMA in the copolymer was determined via integral calculation: the integral ratio of peak e (δ = 7.75 ppm, corresponding to the –NH– protons of DOMA units) to peak h (δ = 4.18 ppm, corresponding to the –OCH_2_– protons of SBMA units), and the results are summarized in Table 1.

As shown in Table 2, with the decrease in DOMA content in the feed formulation, the atomic percentages of O1s and S2p in the DOMA-SBMA copolymers exhibit an increasing trend, while those of C1s and N1s show a decreasing trend. The experimental trend is fully consistent with the theoretical trend, which verifies the variation law of the copolymer’s bulk composition. The theoretical S2p atomic percentages of the DOMA-SBMA_1/9_, DOMA-SBMA_2/8_, and DOMA-SBMA_4/6_ polymers are 3.48%, 4.45%, and 5.05%. Notably, the experimental proportion of S2p in the DOMA-SBMA copolymers is consistently higher than the theoretical value. This is because the catechol groups contained in DOMA tend to interact strongly with the substrate, leading DOMA to preferentially distribute at the coating-substrate interface. As a result, the hydrophilic SBMA zwitterionic groups are pushed toward the coating surface and accumulate there, thus causing the observed deviation in the S2p atomic percentage.

**Figure 2:**
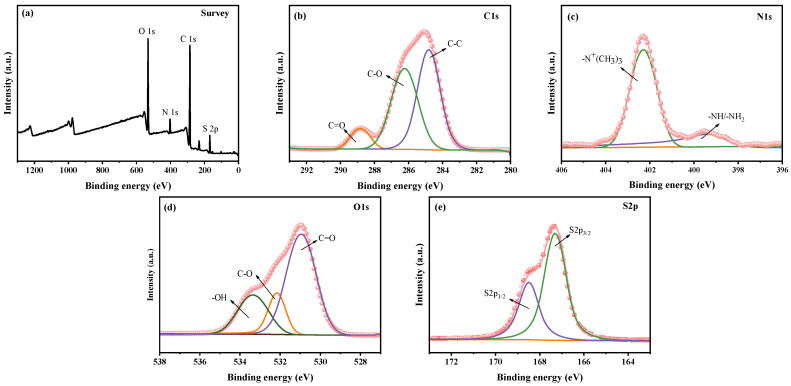
XPS spectra of DOMA-SBMA_2/8_ copolymer (**a**) full spectrum and high-resolution spectra of (**b**) C1s, (**c**) N1s, (**d**) O1s, and (**e**) S2p.

The XPS full spectrum of the stainless steel surface modified with dopamine-based zwitterionic copolymer DOMA-SBMA_2/8_ is shown in Figure 2a. Following modification with the copolymer, a prominent S2p peak emerges on the stainless steel surface, signifying the successful establishment of the zwitterionic polymer coating. The high-resolution spectra of the coating surface elements were peak-fitted using Thermo avantage software. Figure 2b, Figure 2c, Figure 2d and Figure 2e present the comprehensive XPS spectra for C1s, N1s, O1s, and S2p of the DOMA-SBMA_2/8_ coating, respectively. The curve fitting results indicate that the C1s high-resolution spectrum has peaks for C–O bonds at 286.2 eV and C=O bonds at 288.8 eV in the coating. In the N1s high-resolution spectrum, the presence of quaternary ammonium groups results in the peak for –N^+^(CH_3_)_3_ bond at 402.2 eV, with its peak area much larger than that of the –NH/–NH_2_ bond. From the XPS full spectrum, it is evident that a characteristic S peak appears on the surface. The S2p peak exhibits spin–orbit splitting, with S2p_1/2_ and S2p_3/2_ appearing in pairs, and the area of S2p_3/2_ is twice that of S2p_1/2_. In the S2p high-resolution spectrum, peaks at binding energies of 167.1 eV and 168.4 eV are clearly observed for S2p_3/2_ and S2p_1/2_, with the area of S2p_3/2_ being twice of S2p_1/2_. This confirms that the dopamine-based zwitterionic copolymer coating has been successfully prepared, further corroborating the results from ATR-FTIR.

### 3.2. Wettability Characterization

**Figure 3:**
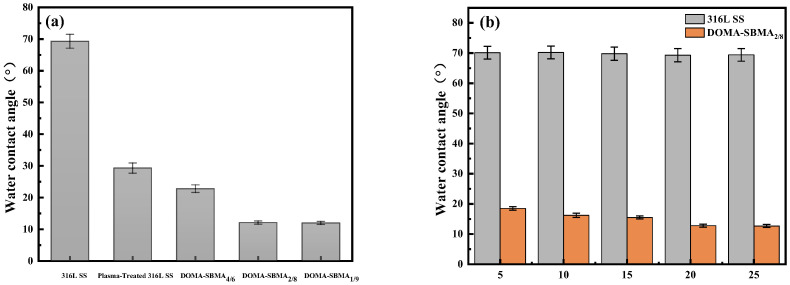
Water contact angles of (**a**) various coatings; (**b**) coatings at different DOMA-SBMA_2/8_ concentrations.

The water contact angle results of samples before and after modification with zwitterionic copolymer coatings at different ratios were measured using a static water contact angle method, as shown in Figure 3a. From the water contact angle results, it can be seen that as the proportion of zwitterionic units in the copolymer increases, the water contact angle on the sample surface gradually decreases. The contact angle on the unmodified 316L stainless steel surface was 69.3 ± 2.2°. After the surface of 316 stainless steel was treated with oxygen plasma, its static contact angle was measured to be 29.3 ± 1.6°, which decreased significantly. This result indicates that a large number of hydroxyl groups (–OH) were generated on the stainless steel surface. After the deposition of the DOMA-SBMA copolymer on the 316L stainless steel surface, the surface hydrophilicity was significantly enhanced: water droplets spread rapidly upon contact with the coated surface, and the zwitterionic surface readily formed a hydration layer with water molecules. As the zwitterionic content on the deposited coating surface increased, the static contact angle value decreased, indicating stronger hydrophilicity. When the molar ratio of DOMA/SBMA was 4:6, the water contact angle on the surface was 22.8 ± 1.2°, and when the molar ratio was 2:8, the contact angle decreased to 12.1 ± 0.5°. Further increasing the SBMA content resulted in negligible changes in the contact angle, although surface hydrophilicity increased with the proportion of zwitterionic units. However, beyond a certain point, the impact on the water contact angle became minimal. The hydrophilic performance of the antibacterial coating is considered one of the key parameters for evaluating its antibacterial properties. Zwitterionic units, relying on their significant superhydrophilic characteristics, can effectively inhibit bacterial adhesion by forming a stable hydration layer through electrostatic interactions with surrounding water molecules. Therefore, the optimal dopamine-based zwitterionic copolymer is DOMA-SBMA_2/8_, which will be used in subsequent experiments.

As seen in Figure 3b, as the concentration of the dopamine-based zwitterionic copolymer increases, the coating water contact angle slightly decreases. When the concentration of the zwitterionic copolymer reaches 5 mg/mL, the surface water contact angle is 18.5 ± 0.6°. When the concentration of the zwitterionic copolymer reaches 20 mg/mL, the surface water contact angle is 12.8 ± 0.5°. Further increasing the copolymer concentration results in minimal changes in the contact angle. This may be due to a slight increase in the reaction rate with higher copolymer concentrations. However, once the concentration reaches a certain level, the effect of concentration change on the reaction rate becomes negligible. Thus, a concentration of 20 mg/mL is chosen for the dopamine-based zwitterionic copolymer.

### 3.3. Stability Analysis

**Figure 4:**
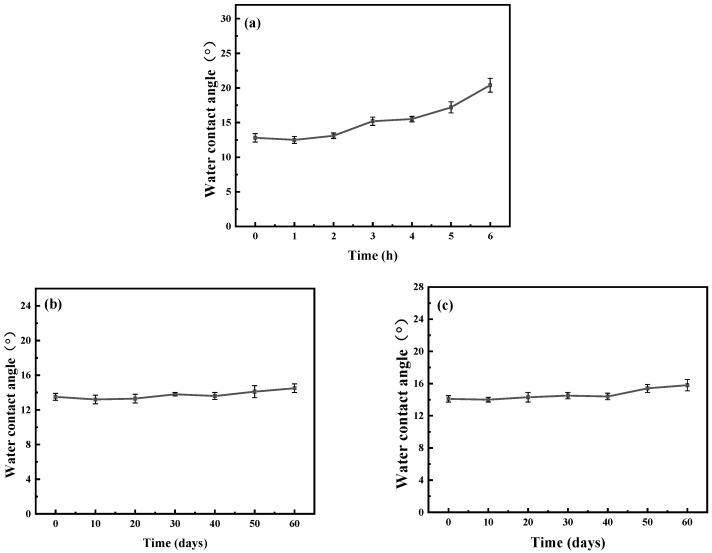
Surface water contact angles of DOMA-SBMA_2/8_ copolymer coatings after (**a**) 6 h of ultrasonic treatment in 75% ethanol, 60 days of immersion (**b**) in PBS solution at pH = 7.4, and (**c**) in 75% ethanol.

Figure 4 shows the changes in the surface water contact angle of the coating under different test conditions. As can be seen in Figure 4a, for the stainless steel sample modified with the DOMA-SBMA_2/8_ copolymer, the water contact angle measures 15.5 ± 0.4° after 5 h of ultrasonic treatment in 75% ethanol, and the corresponding variation in its water contact angle remains less than 5°. The copolymer-modified coating comprises covalent bonds established through Michael addition or Schiff base reactions, in addition to non-covalent bonds such as hydrogen bonds, which collectively enhance the coating’s durability. The dopamine segments within the copolymer are essential for the coating’s durability. The catechol groups of mussel-adhesive dopamine can establish robust chemical interactions with the metal substrate, facilitating a secure adhesion of the coating to the substrate. As shown in Figure 4b and Figure 4c, after immersing the DOMA-SBMA_2/8_ copolymer coating in PBS solution and 75% ethanol solution for 60 days, the surface static water contact angles were measured as 14.5 ± 0.5° and 15.8 ± 0.7°, respectively, with the corresponding variation in the surface static water contact angle being less than 3°. This outstanding stability stems from the covalent binding interactions between the copolymer coating and the stainless steel substrate, demonstrating that the copolymer-modified coating exhibits superior resistance to medium immersion.

### 3.4. Microstructural Analysis

**Figure 5:**
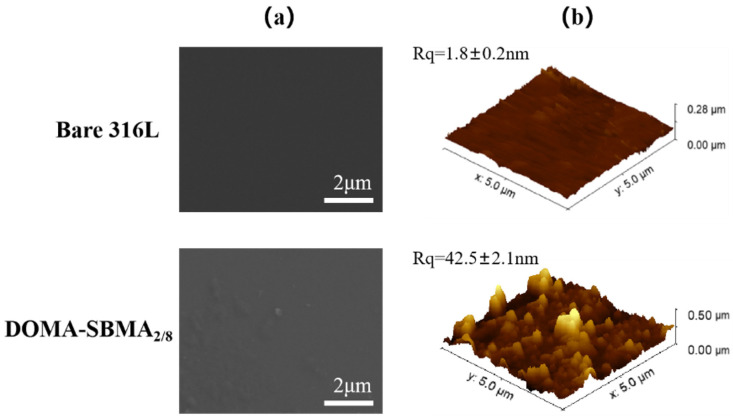
(**a**) SEM images (**b**) AFM 3D images of bare 316L and DOMA-SBMA_2/8_ surfaces.

Figure 5 shows the SEM images and AFM 3D images of the 316L stainless steel surfaces before and after modification. The surface of the unmodified stainless steel is smooth and flat, as shown in Figure 5a. Upon treatment with the dopamine-based zwitterionic copolymer DOMA-SBMA_2/8_, the stainless steel surface exhibits discrete agglomerated particles of irregular size. The formation of these agglomerated particles due to insufficient stirring of the copolymer solution during the reaction process. Since the coating is relatively thin, the number of protruding particles on the coating surface is also relatively small and the copolymer-modified DOMA-SBMA_2/8_ surface remains generally smooth and flat. Figure 5b shows the AFM 3D images of the material surfaces before and after modification. The unmodified stainless steel surface is relatively smooth, with a surface roughness of only 1.8 ± 0.2 nm. After modification with the dopamine-based zwitterionic copolymer DOMA-SBMA_2/8_, the stainless steel surface exhibits protruding particles, the coating gradually thickens, and the surface roughness increases to 42.5 ± 2.1 nm, forming relatively uniform protrusions. This result is consistent with the SEM observations, indicating successful coating formation with good performance.

### 3.5. Analysis of Antibacterial Performance

**Figure 6:**
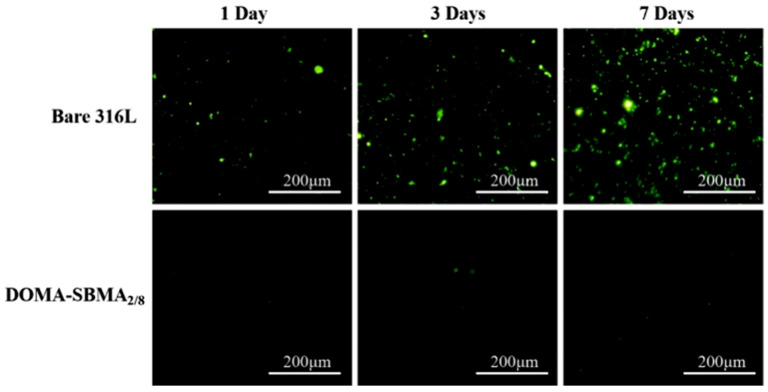
Adhesion images of *S. aureus* at 1 day, 3 days, and 7 days on bare 316L stainless steel and DOMA-SBMA_2/8_-modified surfaces.

**Figure 7:**
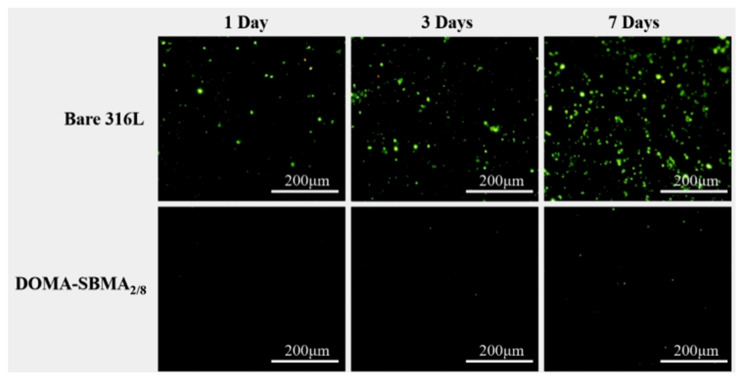
Adhesion images of *E. coli* at 1 day, 3 days, and 7 days on bare 316L stainless steel and DOMA-SBMA_2/8_-modified surfaces.

Figure 6 shows fluorescence microscopy images of the anti-adhesion ability of the modified coating against *S. aureus* before and after modification. The unmodified stainless steel surface exhibited considerable adhesion of *S. aureus* following immersion in the bacterial suspension for a duration, with bacterial adhesion progressively intensifying with time. After one day, a large number of bacteria adhered to the surface; by three days, a substantial quantity had adhered, and by day seven, the surface was nearly entirely covered with bacteria. In contrast, the modified stainless steel surface showed a substantial reduction in bacterial adhesion. As shown in the figure, after incubation in the bacterial suspension for 7 days, only a few bacteria adhered to the surface of the copolymer-modified stainless steel, indicating that the hydrophilic surface displayed strong antibacterial properties, mainly attributed to the hydration effect and steric hindrance caused by the zwitterionic copolymer. This demonstrates that the dopamine-based zwitterionic copolymer coating, DOMA-SBMA_2/8_, can effectively inhibit bacterial adhesion. Figure 7 shows the fluorescence microscope images of the coating’s resistance to *E. coli* adhesion before and after modification. The overall trend is similar to that observed for *S. aureus*. The copolymer coating shows clear resistance to surface colonization by both *S. aureus* and *E. coli*, with a sharp decrease in the number of bacteria.

**Figure 8:**
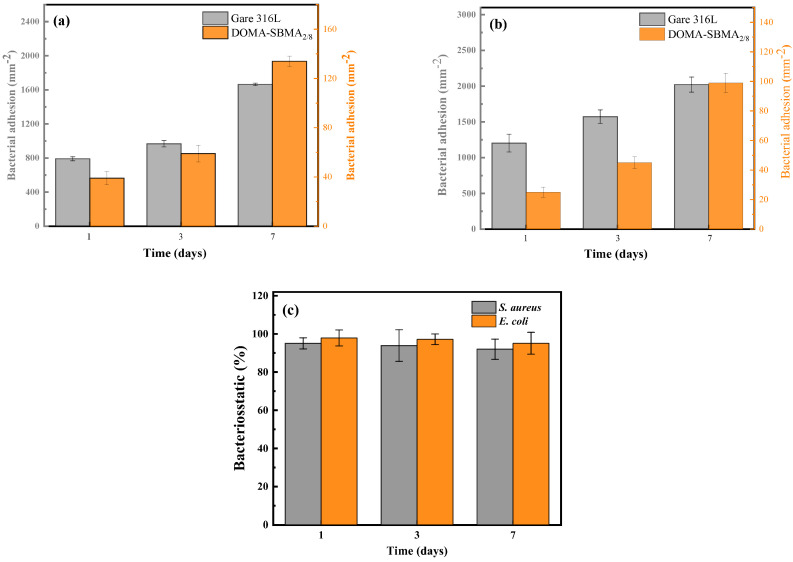
Bacterial adhesion data and antibacterial efficiency of different strains after 1, 3, and 7 days of culture: (**a**) Adhesion data of *S. aureus*; (**b**) Adhesion data of *E. coli*; (**c**) Antibacterial efficiency of DOMA-SBMA_2/8_ coating.

Figure 8 shows the statistical results obtained after analyzing the fluorescence microscope images with ImageJ software. Figure 8a,b depict the changes in bacterial adhesion quantity of untreated 316L stainless steel and DOMA-SBMA_2/8_ copolymer coated samples over 1, 3, and 7 days. As the incubation time increases, the adhesion quantities of *S. aureus* and *E. coli* on the surface of untreated 316L stainless steel both show a significant upward trend. In contrast, the bacterial adhesion quantity on DOMA-SBMA_2/8_ coated samples exhibits a slower upward trajectory. Results of one-way ANOVA indicate that the antibacterial properties between the untreated group and the copolymer coated group exhibit extremely significant differences (*S. aureus*, *p* < 0.05; *E. coli*, *p* < 0.05), confirming that the DOMA-SBMA_2/8_ copolymer coating can effectively inhibit bacterial adhesion and colonization on the stainless steel surface.

Figure 8c further illustrates the antibacterial rate changes of the DOMA-SBMA_2/8_ coating against *S. aureus* and *E. coli*. The coating maintains high antibacterial activity over 1, 3, and 7 days. The sample with dopamine-based zwitterionic copolymer modification exhibited inhibition rates of 94% and 96.9% against *S. aureus* and *E. coli*, respectively, after being cultured in bacterial suspension for 1 day. One-way ANOVA results show that there are significant differences in the antibacterial performance of the DOMA-SBMA_2/8_ coating across different incubation times (*S. aureus*, *p* < 0.05; *E. coli*, *p* < 0.05). After extending the incubation time to 7 days, the inhibition rate on the modified surface for *S. aureus* remained above 90%, and for *E. coli*, it stayed above 95.0%. Although the antibacterial effect of the coating fluctuates slightly with the extension of time, it still remains at an effective antibacterial level overall. This suggests that the dopamine-based zwitterionic copolymer coating has strong hydration ability, excellent antibacterial adhesion performance, and can inhibit the formation of bacterial biofilms. The results indicate that the prepared copolymer DOMA-SBMA_2/8_ effectively inhibits both Gram-positive and Gram-negative bacteria, with a slightly better antibacterial effect against *E. coli* compared to *S. aureus.*

## 4. Conclusions

To address the coating instability issue caused by the strong hydration characteristics of SBMA, this study employs a “grafting-to” strategy to design and synthesize a DOMA-SBMA copolymer on stainless steel substrates. This covalent immobilization approach significantly enhances coating stability while fully preserving the functionality of the zwitterionic segments’ hydrophilic groups, enabling effective antibacterial action through the formation of a hydration layer. The hydrophilicity of the zwitterionic polymer was confirmed by static contact angle measurements, with a surface contact angle of 12.8 ± 0.5°. By optimizing the composition, the DOMA-SBMA composite coating with a DOMA/SBMA molar ratio of 2:8 and DOMA-SBMA concentration of 20 mg/mL was found to possess outstanding performance. Furthermore, the DOMA-SBMA composite coating showed excellent antibacterial properties toward the Gram-negative bacterium *E. coli* and the Gram-positive bacterium *S. aureus*. The DOMA-SBMA composite coating showed excellent adhesion, stability, hydrophilicity, and antibacterial performance, making it promising for applications in marine antifouling and biomedical fields.

## Figures and Tables

**Table 1:** Yield, and molecular weight characteristics of copolymers at different DOMA-SBMA copolymer.

Copolymer	Feed % mol DOMA	Feed % mol SBMA	Polymer Composition DOMA/SBMA	M_n_ (g/mol)	PDI	Yield (%)
DOMA-SBMA_4/6_	40	60	33:67	22,300	1.60	62
DOMA-SBMA_2/8_	20	80	21:79	26,100	1.65	71
DOMA-SBMA_1/9_	10	90	9:91	26,900	1.66	74

**Table 2:** Thickness, elemental composition of DOMA-SBMA copolymer coating.

Copolymer	D (nm)	Atomic Composition (%)			
		C1s	O1s	N1s	S2p
DOMA-SBMA_4/6_	241.2 ± 1.9	67.45	23.31	4.76	4.48
DOMA-SBMA_2/8_	232.1 ± 2.3	64.76	25.03	4.65	5.56
DOMA-SBMA_1/9_	227.8 ± 2.5	63.51	25.82	4.54	6.13

## Data Availability

The original contributions presented in this study are included in the article. Further inquiries can be directed to the corresponding author.

## References

[1] Gu Y.Q., Yu L.Z., Mou J.G., Wu D.H., Xu M.S., Zhou P.J., Ren Y. (2020). Research Strategies to Develop Environmentally Friendly Marine Antifouling Coatings. Mar. Drugs.

[2] Sahoo J., Sarkhel S., Mukherjee N., Jaiswal A. (2022). Nanomaterial-Based Antimicrobial Coating for Biomedical Implants: New Age Solution for Biofilm-Associated Infections. ACS Omega.

[3] Mohandoss S., Velu K.S., Manoharadas S., Ahmad N., Palanisamy S., You S.G., Akhtar M.S., Lee Y.R. (2024). Synthesis, Characterization, and Evaluation of Silver Nanoparticle-Loaded Carboxymethyl Chitosan with Sulfobetaine Methacrylate Hydrogel Nanocomposites for Biomedical Applications. Polymers.

[4] Glinel K., Thebault P., Humblot V., Pradier C.M., Jouenne T. (2012). Antibacterial surfaces developed from bio-inspired approaches. Acta Biomater..

[5] Behzadinasab S., Hosseini M., Williams M.D., Ivester H.M., Allen I.C., Falkinham J.O., Ducker W.A. (2022). Antimicrobial activity of cuprous oxide-coated and cupric oxide-coated surfaces. J. Hosp. Infect..

[6] Chan C.M.N., Cheng H.S., Djurišić A.B., Ng A.M.C., Leung F.C.C., Chan W.K. (2011). Multicomponent antimicrobial transparent polymer coatings. J. Appl. Polym. Sci..

[7] Zhi Z.L., Su Y.J., Xi Y.W., Tian L., Xu M., Wang Q.Q., Padidan S., Li P., Huang W. (2017). Dual-Functional Polyethylene Glycol-b-polyhexanide Surface Coating with In Vitro and In Vivo Antimicrobial and Antifouling Activities. ACS Appl. Mater. Interfaces.

[8] Zhao J.y., Jin S.x., Delgado A.H., Chen Z.f., Matinlinna J.P., Tsoi J.K.H. (2021). Self-Assembled PHMB Titanium Coating Enables Anti-Fusobacterium nucleatum Strategy. Coatings.

[9] Sofroniou C., Scacchi A., Le H., Espinosa Rodriguez E., D’Agosto F., Lansalot M., Dunlop P.S.M., Ternan N.G., Martín-Fabiani I. (2024). Tunable Assembly of Photocatalytic Colloidal Coatings for Antibacterial Applications. ACS Appl. Polym. Mater..

[10] Galvão C.N., Sanches L.M., Mathiazzi B.I., Ribeiro R.T., Petri D.F.S., Carmona-Ribeiro A.M. (2018). Antimicrobial Coatings from Hybrid Nanoparticles of Biocompatible and Antimicrobial Polymers. Int. J. Mol. Sci..

[11] Tang Z.W., Ma C.Y., Wu H.X., Tan L., Xiao J.Y., Zhuo R.X., Liu C.J. (2016). Antiadhesive zwitterionic poly-(sulphobetaine methacrylate) brush coating functionalized with triclosan for high-efficiency antibacterial performance. Prog. Org. Coat..

[12] Zhao C.X., Yuan X.Y., Bai S., Sun P.C., Zhao Y.H., Zhu K.Y., Ren L.X., Li X.H. (2021). Antifogging and antibacterial properties of amphiphilic coatings based on zwitterionic copolymers. Sci. China Technol. Sci..

[13] Yu X.B., Hu S.Y., Zhou Y.H., Yu Z.C., He H.J., Lin S., Long Y. (2025). Green synthesis of electrospun composite material loaded with ultrasmall Ag nanoparticles. Inorg. Chem. Commun..

[14] Li W.T., Zhang Y.F., Ding J.Y., Zhang S., Hu T.Y., Li S., An X.Y., Ren Y.F., Fu Q.W., Jiang X.C. (2022). Temperature-triggered fluorocopolymer aggregate coating switching from antibacterial to antifouling and superhydrophobic hemostasis. Colloids Surf. B.

[15] Guyomard A., Dé E., Jouenne T., Malandain J.J., Muller G., Glinel K. (2008). Incorporation of a Hydrophobic Antibacterial Peptide into Amphiphilic Polyelectrolyte Multilayers: A Bioinspired Approach to Prepare Biocidal Thin Coatings. Adv. Funct. Mater..

[16] Yagci M., Bolca S., Heuts J., Ming W., De With G. (2011). Self-stratifying antimicrobial polyurethane coatings. Prog. Org. Coat..

[17] Ishihara K. (2018). Blood-compatible surfaces with phosphorylcholine-based polymers for cardiovascular medical devices. Langmuir.

[18] Zhang T.L., Liu Q., Meng F.D., Hou Y., Leung M.K., Wen Y.Q., Zhang Q.H. (2024). Recent advances in stimuli-responsive antibacterial coatings: Bacteria-killing and releasing mechanism, design strategies, and potential applications. Prog. Org. Coat..

[19] Zhang M., Yu P., Xie J., Li J.S. (2022). Recent advances of zwitterionic-based topological polymers for biomedical applications. J. Mater. Chem. B.

[20] Ghosh R., Wong W.W., Reimers T., Radzanowski A., Ruiz J.C., Coughlin E.B. (2024). Amphiphilic–zwitterionic block polymers. Polym. Chem..

[21] Yang J., Lin L.G., Tang F.L., Zhao J.Q. (2023). Superwetting membrane by co-deposition technique using a novel N-oxide zwitterionic polymer assisted by bioinspired dopamine for efficient oil–water separation. Sep. Purif. Technol..

[22] Kim I., Kang S.M. (2024). Formation of amphiphilic zwitterionic thin poly (SBMA-co-TFEMA) brushes on solid surfaces for marine antifouling applications. Langmuir.

[23] Li Q.S., Wen C.Y., Yang J., Zhou X.C., Zhu Y.N., Zheng J., Cheng G., Bai J., Xu T., Ji J. (2022). Zwitterionic biomaterials. Chem. Rev..

[24] Zhang H., Li Y., Tian S., Qi X.Y., Yang J., Li Q.S., Lin C.G., Zhang J.W., Zhang L. (2022). A switchable zwitterionic ester and capsaicin copolymer for multifunctional marine antibiofouling coating. Chem. Eng. J..

[25] Xu K.Y., Xie H.M., Sun C.Y., Lin W.Y., You Z.X., Zheng G.C., Zheng X.X., Xu Y.L., Chen J.P., Lin F.C. (2023). Sustainable Coating Based on Zwitterionic Functionalized Polyurushiol with Antifouling and Antibacterial Properties. Molecules.

[26] Zhou R., Ren P.F., Yang H.C., Xu Z.K. (2014). Fabrication of antifouling membrane surface by poly(sulfobetaine methacrylate)/polydopamine co-deposition. J. Membr. Sci..

[27] Chien H.W., Lin H.Y., Tsai C.Y., Chen T.Y., Chen W.N. (2020). Superhydrophilic Coating with Antibacterial and Oil-Repellent Properties via NaIO4-Triggered Polydopamine/Sulfobetaine Methacrylate Polymerization. Polymers.

[28] Ko S., Lee J.Y., Park D., Kim K. (2025). Antimicrobial polymer coatings on surfaces: Preparation and activity. Macromol. Res..

[29] Inoue Y., Onodera Y., Ishihara K. (2018). Initial cell adhesion onto a phospholipid polymer brush surface modified with a terminal cell adhesion peptide. ACS Appl. Mater. Interfaces.

[30] Jiang H.T., Ding K., Meng F.N., Bao L.L., Chai Y.D., Gong Y.K. (2016). Anti-phagocytosis and tumor cell targeting micelles prepared from multifunctional cell membrane mimetic polymers. J. Mater. Chem. B.

[31] Lin X.j., Fukazawa K., Ishihara K. (2015). Photoreactive polymers bearing a zwitterionic phosphorylcholine group for surface modification of biomaterials. ACS Appl. Mater. Interfaces.

[32] Cui Y.X., Yin L.Y., Sun X.Y., Zhang N., Gao N., Zhu G.S. (2021). A Universal and Reversible Wet Adhesive via Straightforward Aqueous Self-Assembly of Polyethylenimine and Polyoxometalate. ACS Appl. Mater. Interfaces.

[33] Liang M., He C.P., Dai J.D., Ren P.F., Fu Y.F., Wang F.M., Ge X., Zhang T.Z., Lu Z.H. (2020). A high-strength double network polydopamine nanocomposite hydrogel for adhesion under seawater. J. Mater. Chem. B.

[34] Wang W., Liu J., Zhu S.L., Wang C.Q., Li Y., Leng X.F. (2025). A Mussel-Inspired Design for Robust Catechol-Functionalized Cross-Linking Adhesives Uniting Water Resistance, Low-Temperature Stability, Recyclability, Degradability, and Fluorescence. ACS Appl. Mater. Interfaces.

[35] Yoon T., Shin M., Yang B., Kim H.J., Lim S., Cha H.J. (2025). Junctional Role of Anionic Domain of Mussel Foot Protein Type 4 in Underwater Mussel Adhesion. Biomacromolecules.

[36] Pal T.S., Raut S.K., Singha N.K. (2025). Mussel-Inspired Catechol-Functionalized EVA Elastomers for Specialty Adhesives; Based on Triple Dynamic Network. Chem. Mater..

[37] Saiz-Poseu J., Mancebo-Aracil J., Nador F., Busqué F., Ruiz-Molina D. (2019). The Chemistry behind Catechol-Based Adhesion. Angew. Chem. Int. Ed..

[38] Tang Z.W., Zhang M., Xiao H., Liu K., Li X.L., Du B.H., Huang L.L., Chen L.H., Wu H. (2022). A Green Catechol-Containing Cellulose Nanofibrils-Cross-Linked Adhesive. ACS Biomater. Sci. Eng..

[39] Ma W.Y., Yang P., Li J.G., Li S.Q., Li P.C., Zhao Y.C., Huang N. (2015). Immobilization of poly (MPC) brushes onto titanium surface by combining dopamine self-polymerization and ATRP: Preparation, characterization and evaluation of hemocompatibility in vitro. Appl. Surf. Sci..

[40] Mu Y.B., Wu X., Pei D.F., Wu Z., Zhang C., Zhou D.S., Wan X.B. (2017). Contribution of the Polarity of Mussel-Inspired Adhesives in the Realization of Strong Underwater Bonding. ACS Biomater. Sci. Eng..

[41] Schneider C.A., Rasband W.S., Eliceiri K.W. (2012). NIH Image to ImageJ: 25 years of image analysis. Nat. Methods.

[42] Wang X.L., Ye Q., Liu J.X., Liu X.J., Zhou F. (2010). Low surface energy surfaces from self-assembly of perfluoropolymer with sticky functional groups. J. Colloid Interface Sci..

